# High prevalence of vitamin D deficiency in Pakistan and miscarriages: A hazard to pregnancies

**DOI:** 10.1016/j.amsu.2022.104634

**Published:** 2022-09-09

**Authors:** Najwa Salim, Muttia Abdul Sattar, Alishba Adnan

**Affiliations:** Karachi Medical and Dental College, Block M North Nazimabad, Karachi, Sindh, 74700, Pakistan

The fact that vitamin D deficiency (VDD) is currently plaguing South Asia and has developed into an endemic condition in that region, is a very troubling situation. Given the significance of vitamin D to one's nutrition, it is exceedingly concerning that Pakistan has the highest incidence of adult vitamin D deficiency in South Asia at 73%, with a mean vitamin D level of 17.93ng/mL [1]. It has the greatest impact primarily on pregnant women in Pakistan, who account for approximately 79.7% of those who do not get enough vitamin D in the general population [2]. This is a cause for considerable concern since it raises the risk of potentially fatal complications during pregnancy, such as gestational diabetes, preeclampsia, and premature births [3].

However, a striking discovery has been made about a complication of pregnancy, miscarriage, that leads to 17% of clinically recognized pregnancies ending in loss [4]. Recently, a review conducted by Jennifer et al. looked at the connection between vitamin D deficiency and miscarriage and made a remarkable conclusion that pregnant women with VDD have a considerably higher risk of miscarriage [5]. Because the converting enzyme, CYP27B1, and the vitamin D receptor(VDR) are widely expressed in decidua and placenta, the human placenta is a significant site for the conversion of inactive 25-hydroxyvitamin D3 [25(OH)-D3] to active 1,25-dihydroxycholecalciferol [1,25(OH)2-D3] [6,7]. There is evidence to indicate that vitamin D has a major impact on both the trophoblastic invasion and the remodelling of the placental artery, both of which are disrupted in a miscarriage [8]. This suggests that a decrease in blood levels of vitamin D can promote placental dysregulation, which in turn might impact the pathophysiology of miscarriage. In addition to this, vitamin D has a valuable role in the mechanism of immunomodulation at the maternal-fetal interface, which contributes to preventing miscarriage even further [9].

Given the prevalence of VDD in pregnant women in Pakistan, this study breakthrough significantly increases the risk of miscarriage for them. There is a high likelihood that VDD and the numerous pregnancy losses in Pakistan are related because it is one of the South Asian nations with the highest burden of pregnancy losses [10].

The country is already being struck by a recent Cholera outbreak amidst the torrential downpours and urban flooding [11] and is still dealing with the impacts of the Coronavirus pandemic. Therefore, there is a dire need to reduce the burden of diseases on the healthcare that is already working at its capacity and stringent measures must be put in place to combat the deficiency, particularly in women of childbearing age. Emphasis should be made on increasing safe exposure to sunlight, as it is the most economical and sustainable source of Vitamin D. As Pakistan is a low-middle-income country, reaching the desired level of vitamin D through diet and supplementation may be a difficult thing due to poverty. The country has a subtropical climate and adequate sunlight throughout the year, yet the majority of the citizens of the country have insufficient serum levels of vitamin D. Women's exposure to sunlight is minimal due to their labor-force force engagement as they are often encouraged to stay at home. Furthermore, there is a deep-rooted obsession in society with fair complexion, which makes women cautious to expose themselves to sunshine. Due to religious and sociocultural norms, most women in Pakistan dress in a way that covers the majority of their skin, restricting their exposure to sunlight.

However, since prolonged exposure to sunlight may lead to the risk of deadly melanomas, in order to address the high deficiency states, public health interventions such as the mandatory fortification of food should be designed and implemented. There is much data suggesting that fortification of frequently consumed foods, such as milk and edibles, may be able to help to an improvement in the vitamin D status of the nations that are located in South Asia [12]. Because of its extensive population coverage and low maintenance expenses, it is a particularly cost-effective choice for addressing deficiencies. Public health programs need to be started in order to create awareness in person and through electronic media. Routine serum 25(OH)-D testing should be incorporated for women of reproductive age, by the healthcare system.

Clinicians and health practitioners should play their part in emphasizing the importance of Vitamin D, promoting its uptake in women of reproductive age and pregnant women and providing them with the correct dosage of supplements to take after assessing their deficient states. As Pakistan has one of the highest percentages of short birth to pregnancy intervals (60%) [13], which can be a great factor contributing to maternal nutritional deficiencies, there is a dire need for family planning programs in order to combat the deficiency of vitamin D in pregnant women.

Most significantly, since there haven't been enough studies on the correlation between miscarriages and VDD, the focus should be placed on more investigations in Pakistan to find such a connection. In addition, there is a need for more research to determine the ideal preconception concentrations of vitamin D that are essential to eliminate the risk of miscarriage caused by a vitamin D deficit.

## Ethical approval

N/A.

## Sources of funding

None.

## Author contributions

Najwa Salim came up with the idea and did submission. Najwa Salim, Muttia Abdul Sattar and Alishba Adnan obtained the data and wrote the article. Muttia Abdul Sattar added the references. Alishba Adnan edited and proof read the article.

## Registration of research studies

1. Name of the registry: N/A
2. Unique Identifying number or registration ID: N/A
3. Hyperlink to your specific registration (must be publicly accessible and will be checked): N/A

## Guarantor

N/A.

## Consent

N/A.

## Declaration of competing interest

None.

## References

[1] Siddiqee M.H., Bhattacharjee B., Siddiqi U.R., MeshbahurRahman M. (2021 Dec). High prevalence of vitamin D deficiency among the South Asian adults: a systematic review and meta-analysis. BMC Publ. Health.

[2] Final Key Findings Report 2019.pdf [Internet]. [cited 2022 Aug 16]. Available from: https://www.unicef.org/pakistan/media/1951/file/Final%20Key%20Findings%20Report%202019.pdf.

[3] De-Regil L.M., Palacios C., Lombardo L.K., Peña-Rosas J.P. (2016 Jan 14). Vitamin D supplementation for women during pregnancy. Cochrane Database Syst. Rev..

[4] Ventura S.J., Curtin S.C., Abma J.C., Henshaw S.K. (2012 Jun 20). Estimated pregnancy rates and rates of pregnancy outcomes for the United States, 1990-2008. Natl Vital Stat Rep Cent Dis Control Prev Natl Cent Health Stat Natl Vital Stat Syst.

[5] Tamblyn J.A., Pilarski N.S.P., Markland A.D., Marson E.J., Devall A., Hewison M. (2022 Jul). Vitamin D and miscarriage: a systematic review and meta-analysis. Fertil. Steril..

[6] Weisman Y., Harell A., Edelstein S., David M., Spirer Z., Golander A. (1979 Sep 27). 1 alpha, 25-Dihydroxyvitamin D3 and 24,25-dihydroxyvitamin D3 in vitro synthesis by human decidua and placenta. Nature.

[7] Zehnder D., Evans K.N., Kilby M.D., Bulmer J.N., Innes B.A., Stewart P.M. (2002 Jul 1). The ontogeny of 25-hydroxyvitamin D3 1α-hydroxylase expression in human placenta and decidua. Am. J. Pathol..

[8] Chan S.Y., Susarla R., Canovas D., Vasilopoulou E., Ohizua O., McCabe C.J. (2015 Apr 1). Vitamin D promotes human extravillous trophoblast invasion in vitro. Placenta.

[9] Tamblyn J.A., Hewison M., Wagner C.L., Bulmer J.N., Kilby M.D. (2015 Mar 1). Immunological role of vitamin D at the maternal–fetal interface. J. Endocrinol..

[10] Lawn J.E., Blencowe H., Waiswa P., Amouzou A., Mathers C., Hogan D. (2016 Feb 6). Stillbirths: rates, risk factors, and acceleration towards 2030. Lancet Lond Engl.

[11] Taseen S., Jawed S., Mohammad Ameen A. (2022 Aug 18). Cholera outbreak amidst urban flooding in Karachi: an emergency condition. Ann Med Surg.

[12] Yang Z., Laillou A., Smith G., Schofield D., Moench-Pfanner R. (2013 Jun 1). A review of vitamin D fortification: implications for nutrition programming in southeast Asia. Food Nutr. Bull..

[13] Nausheen S., Bhura M., Hackett K., Hussain I., Shaikh Z., Rizvi A. (2021 Apr 26). Determinants of short birth intervals among married women: a cross-sectional study in Karachi, Pakistan. BMJ Open.

